# Supplementary material for: Mapping the evolution of fertility support policies in China: A content and instrumental analysis
Source: PLoS One. 2025 Oct 9;20(10):e0332137. doi: 10.1371/journal.pone.0332137 (PMC12510515; doi:10.1371/journal.pone.0332137)
Supplement: S1 Appendix — (ZIP) [file pone.0332137.s001.zip › S1 Appendix. 226 original policy documents/70-国务院关于就业工作情况的报告(FBM-CLI.2.5146799).doc]

**国务院关于就业工作情况的报告**

制定机关： [国务院](https://www.pkulaw.com/law/chl?Aggs.IssueDepartment=5&way=textBasic)

公布日期：2022.12.28

施行日期：2022.12.28

时效性： [现行有效](https://www.pkulaw.com/law/chl?Aggs.TimelinessDic=01&way=textBasic)

效力位阶： [国务院规范性文件](https://www.pkulaw.com/law/chl?Aggs.EffectivenessDic=XC0203&way=textBasic)

法规类别： [招工与就业](https://www.pkulaw.com/law/chl?Aggs.Category=08702&way=textBasic) [营商环境优化](https://www.pkulaw.com/law/chlhttps://dobusiness.pkulaw.com)

国务院关于就业工作情况的报告
--2022年12月28日在第十三届全国人民代表大会常务委员会第三十八次会议上
人力资源和社会保障部副部长　俞家栋

全国人民代表大会常务委员会：
　　我受国务院委托，向全国人大常委会报告就业工作情况，请审议。
　　党中央、国务院高度重视就业工作。党的二十大对就业工作作出重要部署。习近平总书记强调，就业是最基本的民生，要实施就业优先战略，强化就业优先政策，健全就业公共服务体系，加强困难群体就业兜底帮扶，消除影响平等就业的不合理限制和就业歧视，使人人都有通过勤奋劳动实现自身发展的机会。李克强总理指出，要通过稳市场主体保就业，更多用市场化社会化办法增加就业岗位，抓好高校毕业生、农民工等重点群体就业。栗战书委员长专门对做好[就业促进法](https://www.pkulaw.com/chl/1e8e09abdbcff012bdfb.html?way=textSlc)执法检查作出重要批示，明确要求为促进就业工作提供法治保障。人力资源社会保障部会同有关方面认真贯彻落实党中央、国务院决策部署，全力以赴推进就业各项工作，实现就业局势总体稳定。
　　**一、**新时代十年我国就业工作取得显著成效
　　党的十八大以来，我国深入实施就业优先战略，先后印发两个就业促进五年规划，有关部门认真落实各项部署，主动应对风险挑战，推动就业工作取得显著成效。十年来，就业规模持续扩大，就业结构不断优化，就业质量稳步提升，重点群体就业保持稳定，在14亿多人口的大国实现了比较充分的就业，为经济发展和民生改善提供了重要支撑。
　　（一）就业优先导向更加鲜明。强化方向引领，将就业作为“六稳”、“六保”的首要任务，在推动经济高质量发展中促进就业扩容提质。一是创新实施就业优先的宏观调控。首次提出就业优先政策并置于宏观政策层面，把城镇新增就业、城镇调查失业率作为经济社会发展的主要预期目标，作为制定实施宏观政策的优先考量，不断提高经济发展就业带动力。2012年以来，全国城镇新增就业平均每年超过1300万人，2018年以来全国城镇调查失业率平均在5.2%左右。二是优化推进就业导向的结构调整。稳步实施区域重大战略和区域协调发展战略，中西部地区劳动者就地就近就业和返乡创业明显增多，区域就业结构更趋合理。全力拓宽农民增收致富渠道，加快推进以人为核心的新型城镇化，城乡就业结构发生历史性变化，2013年城镇就业比重首次超过乡村，2021年达到62.7%。积极建设现代化产业体系，提升农业就业质量，稳定制造业就业规模，大力发展就业带动能力强的服务业，三次产业就业结构与产值结构的协调性明显提高，第三产业从业人员占比从2012年的36.1%提升到2021年的48.0%。三是加快健全就业协同的责任机制。就业工作部际联席会议升格为国务院就业工作领导小组，将城镇调查失业率纳入高质量发展综合绩效评价体系，将就业政策落实作为财政支持的重要方向，接续开展[就业促进法](https://www.pkulaw.com/chl/1e8e09abdbcff012bdfb.html?way=textSlc)执法检查、就业工作专项督查、就业工作表扬激励。
　　（二）就业政策体系更加完善。坚持问题导向，构建完善就业优先政策体系，精准发力支持稳就业保就业。一是加力实施减负稳岗系列政策。打出社会保险“降免缓返补”政策组合拳。先后7次降低社会保险费率，5项社会保险单位费率由2012年的41%降至目前的33.95%；2020年疫情期间出台力度空前的阶段性社会保险费减免政策，累计减免相关社会保险费1.54万亿元；2022年对部分特困行业和困难企业阶段性缓缴养老、失业、工伤三项社会保险费，对中小微企业阶段性缓缴职工医保单位缴费，截至11月底共缓缴2052亿元；加大失业保险稳岗返还政策力度，截至11月底累计返还484亿元，惠及769万户企业；创新推出一次性留工培训补助，截至11月底已发放259亿元，惠及472万户企业；出台住房公积金阶段性支持政策，截至11月25日，全国共支持2.92万户企业缓缴住房公积金103亿元。二是发挥财政职能作用丰富拓展扩岗激励举措。2012年以来，中央财政累计安排就业补助资金预算5344亿元，支持地方实施社会保险补贴、职业培训补贴、就业见习补贴、吸纳就业补贴等就业扶持政策，并扩大实施范围，合理确定补贴标准。此外，通过税收减免、以工代训、留工培训补助等政策，鼓励企业扩大用工、吸纳就业。三是加快构建灵活就业、新就业形态服务和保障制度。首次出台支持灵活就业健康发展专门意见，持续细化配套举措。完善网约配送员、网约车驾驶员、网约货车司机等新就业形态劳动者权益保障制度，督促平台企业依法合规用工；放开灵活就业人员在就业地参加基本养老保险的户籍限制，支持自主选择缴费方式、缴费基数，落实困难人员灵活就业社会保险补贴政策；启动新就业形态劳动者职业伤害保障试点，制定业务经办和征收管理规程，推进灵活就业人员参加住房公积金制度试点；出台加强零工市场建设意见，建立“即时快招”服务模式，鼓励市场机构开展零工服务，引导自发市场规范发展，要求公共就业服务机构开设零工专区，推动建设零工市场超3200家，日均提供对接服务超27万人次。
　　（三）重点群体就业稳步推进。突出重点难点，分类帮扶、精准施策，兜住兜牢重点群体就业底线。一是坚定不移把高校毕业生等青年就业作为重中之重。国务院每年召开专题会议部署推动，连续出台政策加力支持。相关部门、社会各界聚力攻坚，自上而下成立工作专班，全力拓宽市场化就业渠道，大力挖潜基层就业空间，稳定扩大公共部门岗位规模。搭建国家大学生就业服务平台、公共招聘网等青年就业服务平台，持续实施青年百万见习计划，深入开展高校毕业生“三支一扶”计划、教师特岗计划和西部计划等基层服务项目，创新开展科研助理岗位开发和落实工作，持续开展百日千万、书记校长访企拓岗、国聘行动、中小企业网上百日招聘高校毕业生、女大学生专场活动、阳光就业暖心行动等招聘活动，实施“宏志助航”、一般院校低收入家庭学生就业帮扶等计划，加强困难毕业生专项帮扶和离校未就业毕业生实名帮扶，确保工作不断档、服务不断线。2012年以来，我国高校毕业生年底总体就业率保持在90%以上。二是全力稳定农村劳动力特别是脱贫劳动力务工规模。坚持有序外出和就地就近就业相结合，完善就业服务、职业培训、权益保障支持体系，推动劳务协作扩容提质，加快劳务品牌培育发展，搭建劳动者返乡入乡创业支持平台，推动在重点工程项目、农业农村基础设施建设领域中大力实施以工代赈，创新开展农村劳动力就业大数据监测分析。疫情期间创新开展“点对点”返岗服务、“稳岗留工”等专项行动，全力保障劳动者就业增收。脱贫劳动力务工规模从2015年的1527万人增至2022年11月份的3270万人，2/3的脱贫家庭实现了务工就业，务工就业收入占到脱贫家庭收入的2/3。三是不断健全困难人员就业援助制度。建立日常援助和集中援助相结合的工作格局，形成及时发现、及时认定、及时帮扶的工作机制，完善公益性岗位规范管理办法，持续开展“就业援助月”、“暖心活动”等专项行动，全力兜住困难人员底线。2012年以来，累计帮助失业人员再就业5977万人，就业困难人员就业1931万人，城乡残疾人每年新增就业超30万人，实现零就业家庭至少一人就业。四是有效保障其他重点群体就业。深化退役军人安置制度改革，健全政策扶持体系，强化学历提升、技能培训，拓宽民营企业、乡村振兴、基础教育、自主创业等就业渠道。做好重大改革涉及人员安置工作，140万去产能职工得到妥善安置，截至2022年三季度末，16万长江流域退捕渔民实现转产就业。加强对生育中断再就业妇女的帮扶。五是扎实做好失业人员基本生活保障。实施失业保障扩围政策，阶段性扩大价格临时补贴受益范围至领取失业补助金人员，对距法定退休年龄不足1年的失业人员继续发放失业保险待遇至退休，为女性失业人员增加代缴生育保险费。2012年以来，累计为8095万人次发放失业保险待遇5550亿元。
　　（四）创业带动效应加快释放。坚持创新驱动，激发劳动者创业潜能，充分发挥创业带动就业的倍增效应。一是深化改革优环境。国务院连续10年召开深化“放管服”改革会议，深入推进商事制度改革，实施[市场准入负面清单](https://www.pkulaw.com/chl/0034c9a92059f448bdfb.html?way=textSlc)管理，压减企业开办时间，出台试行《[促进个体工商户发展条例](https://www.pkulaw.com/chl/90daa82a62ac3943bdfb.html?way=textSlc)》，提供便企利民政务服务，不断优化营商环境，截至11月底，登记在册市场主体达到1.68亿户，其中个体工商户1.13亿户，比十年前增长2倍，支撑稳住了就业基本盘。二是强化扶持添动力。开展创新创业教育，加大创业培训力度，提供政策咨询、开业指导、场地支持、跟踪扶持等一条龙创业服务，打造创业孵化基地8800多家、众创空间8500多家、孵化器5800多家，服务创业团队和企业超过65.8万家，年均提供创业培训超200万人次、创业服务超400万人次。拓宽创业融资渠道，持续加大创业担保贷款贴息力度，将中央财政贴息支持的个人创业担保贷款额度上限由10万元增至20万元，小微企业创业担保贷款额度上限由200万元增至300万元，放宽贷款申请条件和反担保要求，简化贷款申请流程，创业担保贷款余额由2012年末的788亿元增至2022年三季度末的2694亿元。三是营造氛围激活力。连续8年成功举办全国“大众创业万众创新活动周”，持续实施创业带动就业示范行动，精心组织“互联网＋”、中国创翼、创客中国、创青春、农村双创项目创意、挑战杯等赛事活动，创新创业热潮蓬勃兴起。
　　（五）就业服务体系日益健全。顺应群众期盼，扩大服务供给，创新运行机制，打造覆盖全民、贯穿全程、辐射全域、便捷高效的全方位公共就业服务。一是确立基本公共服务制度。强化常住地免费服务制度，面向全体劳动者和各类用人单位提供职业介绍、职业指导、政策咨询等基本公共服务。持续组织“10＋N”公共就业服务专项活动，创新实施重点企业用工服务保障机制。2012年以来，年均为各类市场主体和劳动者提供免费基本公共服务超2亿人次。二是构建多元化供给体系。建立以城市为中心、覆盖城乡的公共就业服务体系，完善公共就业服务机构设置，统筹优化综合性服务场所和人力资源市场、零工市场等专业化服务场所。强化经营性人力资源服务机构管理创新，优化人力资源服务行政许可、审批流程，鼓励外商投资人力资源服务，分类培育综合性人力资源服务企业和专精特新人力资源服务企业、单项冠军企业。支持工青妇、行业协会商会等人民团体、社会组织，结合自身业务范围和专长，依法有序提供就业服务。截至2021年底，各类人力资源服务机构达5.91万家，营业收入超过2.46万亿元。三是全面提升就业服务质量。实施提升就业服务质量工程，启动公共就业服务示范项目，扩大就业失业管理服务覆盖范围，加强重大任务专项保障能力。推进“互联网＋”公共就业服务，加快大数据、云服务技术运用，创新云招聘、远程面试、直播带岗等服务模式，建成失业登记平台、中国公共招聘网等智能公共就业服务平台，公共招聘服务平台日均活跃岗位信息超千万个，线上线下一体化就业服务格局基本形成。
　　（六）职业培训制度不断完善。适应发展需要，完善政策制度，实施专项计划，打造规模宏大、结构合理的高技能人才队伍。一是健全终身职业技能培训制度。完善职业技能培训政策体系，开展常态化大规模多层次职业技能培训，稳步实施重点群体专项培训计划，支持企业开展职工在岗培训，突出技能人才培训、急需紧缺人才培训、转岗转业培训、储备技能培训、通用职业素质培训，广泛开展高素质农民技能培训，完善职业技能竞赛体系。从失业保险基金结余中拿出超1000亿元实施职业技能提升三年行动，开展补贴性职业技能培训8300多万人次，劳动者稳定就业和转换岗位能力不断增强。二是提升职业技能培训质量。健全职业技能培训工作协调机制，构建以行业企业为主体、培训机构和职业学校（含技工院校）为基础、政府推动与社会支持相结合的职业技能培训组织实施体系，健全职业技能培训共建共享机制，引导培训资源向市场急需、企业生产必需等领域集中。规范开展订单式培训，健全培训监督评价考核机制，增强培训针对性和实效性。2016年至2021年，中央预算内投资累计投入55.5亿元，支持建设公共实训基地216家。三是完善技能人才培养、使用、评价和激励机制。推进职业资格制度改革，完善职业技能等级制度，推行社会化职业技能等级认定，发布新版国家职业分类大典，增加158个新职业。畅通技能人才职业发展通道，完善技能人才薪酬、表彰等激励政策，启动特级技师评聘试点，健全完善新时代技能人才职业技能等级制度。截至2021年底，全国技能劳动者总量增至2亿人以上，其中高技能人才超过6000万。
　　（七）劳动者合法权益有效维护。完善制度机制，提升治理效能，切实维护劳动者合法权益，不断提升就业质量。一是完善劳动关系协商协调机制。健全劳动法律法规体系，深入实施劳动关系“和谐同行”能力提升三年行动计划，提升劳动关系治理能力。完善协调劳动关系三方机制，深入推进和谐劳动关系创建活动，建立规范有序、公正合理、互利共赢、和谐稳定的和谐劳动关系。二是改善劳动者就业条件。健全工资决定、合理增长和支付保障机制，完善最低工资标准调整机制，增加劳动者特别是一线劳动者劳动报酬。2021年，城镇非私营单位和私营单位就业人员平均工资比2012年分别实际增长90.8%、82.7%，超过同期劳动生产率增幅。持续推进全民参保计划，稳步提高社会保险统筹层次和待遇水平，完善全国统一的社会保险公共服务平台，优化社会保险关系转移接续，建成世界上规模最大的社会保障体系。三是规范企业用工。加强劳动争议预防和调处，做好对劳动密集型企业、中小微企业用工指导。强化劳动保障监察执法，依法查处招聘过程中的虚假招聘等违法行为，及时纠正性别、年龄及针对新冠肺炎康复者的就业歧视，开展就业性别歧视联合约谈和妇女平等就业权检察公益诉讼。

　　**二、**就业工作面临的总体形势
　　当今世界正经历百年未有之大变局，我国人口结构、经济社会发展等正发生深刻变化，就业工作既面临错综复杂环境变化带来的新问题新挑战，也迎来高质量发展和社会主要矛盾变化带来的新机遇新要求。
　　党的二十大擘画了以中国式现代化全面推进中华民族伟大复兴的使命任务，明确了未来五年是全面建设社会主义现代化国家开局起步的关键时期，作出了增进民生福祉、提高人民生活品质的重要部署，明确提出实施就业优先战略，强化就业优先政策，为实现高质量充分就业提供了根本遵循。我国已进入高质量发展阶段，新发展格局加快构建，经济韧性强、潜力大、活力足、长期向好的基本面没有变，新的就业增长点将不断涌现，为保持就业局势长期稳定提供了坚实基础。劳动力市场协同性增强，劳动者受教育程度继续上升，社会性流动更加顺畅，为推进就业扩容提质提供了有力支撑。
　　与此同时，当前我国经济恢复的基础尚不牢固，新的影响因素不断增加，劳动力市场固有矛盾依然存在，就业工作仍面临不少风险挑战。
　　（一）就业总量压力依然不减。我国是人口和劳动力大国，2021年末16-59岁劳动年龄人口8.8亿人，“十四五”期间仍将保持在8.5亿人以上，超过欧洲人口的总和。与此同时，经济发展不确定性依然较大，需求收缩、供给冲击、预期转弱三重压力仍然较大，稳定就业存量、扩大就业增量面临较大压力，保持充分就业始终是首要任务。
　　（二）结构性就业矛盾日益凸显。劳动力供给和需求存在错位，招工难与就业难并存的结构性就业矛盾成为就业领域的主要矛盾。一方面，企业招工难问题突出，服务员、操作工等一线普工常年短缺，技能人才供给不足；另一方面，产业转型升级加快，部分劳动者技能转换需要时间，求职就业难度或将进一步加大。
　　（三）部分重点群体就业面临难题。“十四五”期间，16-24岁青年人口将达到1.4亿左右，其中每年高校毕业生总量超过千万，专业、区域、意愿等结构性矛盾短期难以缓解，青年失业水平仍将高位运行。人口老龄化加深，大龄劳动者就业竞争力偏弱，市场求人倍率长期在1以下。黑中介、假招聘等违法违规问题时有发生，年龄、性别、残疾等就业歧视依然存在，女性劳动者失业水平仍将高企，新就业形态劳动者权益维护任务依然艰巨。
　　（四）疫情等因素对就业造成的影响尚需恢复。受疫情因素影响，叠加世界经济复苏乏力，市场预期不稳，企业缩招减员增多，城镇新增就业下滑，失业水平上升。当前，就业容量大的批发零售、住宿餐饮、交通旅游等行业和受疫情冲击地区恢复还需要时间，部分外贸出口企业受外部环境影响复苏仍然缓慢，就业增长、失业控制的压力短期内仍然较大。
　　（五）就业促进机制有待完善。就业优先导向仍需进一步落地，高质量充分就业评价体系亟待建立，有利于稳定和扩大就业的宏观调控措施、产业结构布局有待强化，重大政策、重大工程、重大项目实施对就业的影响评估机制有待健全。面对就业方式多元化、就业需求多样化、就业流动常态化的趋势，现行劳动就业、社会保障管理服务制度仍需加快调整完善。
　　（六）就业服务培训维权工作体系有待加强。公共就业服务基层基础仍然薄弱，专业化队伍建设亟待加强，信息化管理服务水平有待提高，全国统一、共建共享、高效联通的公共就业服务信息平台需进一步集成完善。职业培训能力建设有待加强，适应发展需求、符合就业导向的终身职业技能培训制度有待完善。劳动保障执法监察、劳动人事争议调解仲裁力量不足，案多人少矛盾突出，基层力量亟待强化，就业性别歧视联合约谈力度仍需加强，劳动者权益维护任务依然繁重。

　　**三、**下一步工作考虑
　　以习近平新时代中国特色社会主义思想为指导，全面贯彻落实党的二十大精神，落实党中央、国务院决策部署，按照全国人大常委会审议意见要求，坚持以人民为中心的发展思想，以实施就业优先战略为引领，以高质量充分就业为目标，立足基本国情，把握发展规律，注重市场引领、政府引导，坚持做好稳就业保就业工作，强政策、优服务、保权益，激发全社会就业创业活力，多措并举促进就业扩容提质、合理公平、畅通有序，全力以赴保持就业局势总体稳定，为扎实推进共同富裕、拓展中国式现代化贡献就业力量。
　　（一）全面强化就业优先政策。强化就业优先导向的宏观调控，实施有利于扩大就业的财政、货币政策，推动投资、消费、产业、区域等政策与就业政策衔接配套。打造就业友好型现代化产业体系，促进农业就业增收、制造业就业提质、服务业就业扩容，加大对就业容量大的民营企业、中小微企业和个体工商户支持力度。健全就业促进机制，修订[就业促进法](https://www.pkulaw.com/chl/1e8e09abdbcff012bdfb.html?way=textSlc)，制定高质量充分就业支持政策和评价体系，建立公共投资和重大项目带动就业评估机制。
　　（二）完善重点群体就业支持体系。把促进青年特别是高校毕业生就业工作摆在更加突出的位置，对接国家重大战略实施、产业优化布局、区域协调发展和重点行业需求拓宽就业渠道，实施“高校毕业生就业创业促进行动”，推动公共就业服务更多向高校毕业生等青年就业群体倾斜，促进政策广覆盖、服务不断线、就业水平有提升。推进农村劳动力转移就业特别是脱贫人口稳定就业，统筹城乡就业政策体系，通过深化劳务协作、培育劳务品牌引导有序外出，依托乡村产业、县域经济发展、农村基础设施建设支持就地就近就业，加大以工代赈投资力度，尽可能增加劳务报酬发放规模，健全脱贫人口、农村低收入人口就业帮扶长效机制。加强城乡困难群体援助帮扶，构建大龄、残疾等就业困难人员及时发现、优先服务、分类帮扶、动态管理机制。统筹做好退役军人、女性等其他群体就业工作。
　　（三）促进创业带动就业和灵活就业。优化创业环境，推进行政审批制度改革，落实[市场准入负面清单](https://www.pkulaw.com/chl/0034c9a92059f448bdfb.html?way=textSlc)制度，持续清理对个人经营的不合理限制。加强政策服务支持，实施重点群体创业推进行动，加大贷款贴息、创业补贴、场地支持、租金减免等扶持力度，用足用好相关税收优惠政策，强化创业载体建设，大力促进科技创业带动高质量就业，举办中国创翼、“互联网＋”大学生创新创业大赛等赛事交流活动。加强新就业形态劳动者权益保障，完善零工市场建设举措，明确平台企业的劳动者权益保障责任，继续推进新就业形态劳动者职业伤害保障试点。
　　（四）健全就业公共服务体系。推动就业公共服务扩面提质，持续推进提升就业服务质量工程，加强就业服务人员队伍建设，以创建示范项目、示范城市、示范社区为抓手，以打造活动品牌、招聘平台、基层网点、零工市场为载体，以数字化为牵引，不断健全公益性、兜底性就业公共服务体系，提高公共服务水平，增强均衡性和可及性。推动人力资源服务业高质量发展，实施人力资源服务行业促就业行动，优化市场准入，完善法规制度，注重规范监管，加强龙头企业培育和国家级产业园建设。推动相关公共服务系统协同，探索完善就业配套公共服务体系，推动劳动者在常住地、就业地均等享受基本教育、医疗、养老、住房等公共服务。
　　（五）健全终身职业技能培训制度。落实[加强新时代高技能人才队伍建设意见](https://www.pkulaw.com/chl/c00d0e291e42ede3bdfb.html?way=textSlc)，推进“技能中国行动”，加强先进制造业、数字技能等重点领域高技能人才培养力度，广泛开展养老护理、家政服务等康养领域和手工、电商等新业态领域就业技能培训，建设一批集技能培训、技能评价、技能竞赛、技能交流、工匠精神传播等为一体的综合型高技能人才培养培训载体，支持各类市场主体依法设立职业培训机构，推动公共实训基地共建共享，促进职业培训提质增效。健全技能人才岗位使用、合理流动机制，完善技能人才薪酬分配制度，建立技能人才多元化评价机制，推行“新八级工”职业技能等级（岗位）序列，健全职业标准体系和评价制度，畅通技能人才职业发展通道。举办全国技能大赛、全国乡村振兴职业技能大赛等赛事活动。
　　（六）完善劳动权益保障制度。破除妨碍影响劳动力、人才流动的体制和政策弊端，消除影响平等就业的不合理限制和就业歧视，研究制定平等就业综合性法规政策，完善就业性别歧视联合约谈机制，加大对性别、年龄以及新冠康复者等就业歧视现象的纠治力度。完善工资收入分配制度，加强企业工资收入分配宏观调控和指导服务，保障劳动者合法报酬权益。加强劳动争议调处，强化人力资源市场监管和劳动保障监察执法。防范就业领域风险，健全就业失业监测预警机制和风险应对处置机制，完善规模失业风险应对预案。
　　委员长、各位副委员长、秘书长、各位委员，长期以来，全国人大常委会高度重视、大力支持就业工作，加强就业领域立法和执法监督，有力推动了民生就业发展。我们将在以习近平同志为核心的党中央坚强领导下，深刻领悟“两个确立”的决定性意义，增强“四个意识”、坚定“四个自信”、做到“两个维护”，切实把思想认识行动统一到习近平总书记关于就业工作的重要讲话和指示批示精神上来，坚定不移贯彻落实党中央、国务院决策部署，按照本次会议审议提出的意见，积极采取有效举措，推动实现高质量充分就业，为推动我国经济高质量发展提供有力支撑。

#### 本篇引用的法规

##### 中央法规

[中共中央办公厅、国务院办公厅印发《关于加强新时代高技能人才队伍建设的意见》](https://www.pkulaw.com/chl/c00d0e291e42ede3bdfb.html?way=textRightFblx)

[促进个体工商户发展条例](https://www.pkulaw.com/chl/90daa82a62ac3943bdfb.html?way=textRightFblx)

[国家发展改革委、商务部关于印发《市场准入负面清单(2020年版)》的通知](https://www.pkulaw.com/chl/0034c9a92059f448bdfb.html?way=textRightFblx)

[中华人民共和国就业促进法(2015修正)](https://www.pkulaw.com/chl/1e8e09abdbcff012bdfb.html?way=textRightFblx)

©北大法宝：（[www.pkulaw.com](https://www.pkulaw.com/)）专业提供法律信息、法学知识和法律软件领域各类解决方案。北大法宝为您提供丰富的参考资料，正式引用法规条文时请与标准文本核对。 欢迎查看所有[产品和服务](http://www.pkulaw.net/)。
[法宝快讯： 如何快速找到您需要的检索结果？ 法宝 V6 有何新特色？](http://www.pkulaw.com/helps/69.html)

扫描二维码阅读原文

原文链接：<https://www.pkulaw.com/chl/1a20bf1a80547d30bdfb.html>
